# Malformin-A1 (MA1) Sensitizes Chemoresistant Ovarian Cancer Cells to Cisplatin-Induced Apoptosis

**DOI:** 10.3390/molecules26123624

**Published:** 2021-06-13

**Authors:** Nada Abdullah, Yahya Tamimi, Sergey Dobretsov, Najwa Al Balushi, Jalila Alshekaili, Hamed Al Balushi, Mahmood Al Kindi, Syed Imran Hassan, Shadia Al Bahlani, Benjamin K. Tsang, Ikram A. Burney

**Affiliations:** 1 Department of Biochemistry, College of Medicine & Health Sciences, Sultan Qaboos University, P.O. Box 50, Muscat P.C. 123, Oman; nadaabdulla111@gmail.com (N.A.); n.km@windowslive.com (N.A.B.); 2 Department of Marine Science & Fisheries, College of Agricultural & Marine Sciences, Sultan Qaboos University, P.O. Box 50, Muscat P.C. 123, Oman; sergeydobretsov@gmail.com; 3 Centre of Excellence in Marine Biotechnology, Sultan Qaboos University, P.O. Box 50, Muscat P.C. 123, Oman; 4 Department of Microbiology and Immunology, Sultan Qaboos University Hospital, Sultan Qaboos University, P.O. Box 50, Muscat P.C. 123, Oman; jalila@squ.edu.om (J.A.); hamadtaj20@gmail.com (H.A.B.); dralkindi@squ.edu.om (M.A.K.); 5 Department of Chemistry, College of Science, Sultan Qaboos University, P.O. Box 50, Muscat P.C. 123, Oman; s.hasan@squ.edu.om; 6 Department of Allied Health Sciences, College of Medicine & Health Sciences, Sultan Qaboos University, P.O. Box 50, Muscat P.C. 123, Oman; bahlani@squ.edu.om; 7 Departments of Obstetrics & Gynecology, Cellular & Molecular Medicine and the Interdisciplinary School of Health Sciences and the Centre for Infection, Immunity and Inflammation, Chronic Disease Program, Ottawa Hospital Research Institute, University of Ottawa, Ottawa, ON K1N 6N5, Canada; btsang@ohri.ca; 8 Department of Medicine, College of Medicine & Health Sciences, Sultan Qaboos University, P.O. Box 50, Muscat P.C. 123, Oman; ikram@squ.edu.om; 9 Panjwani Center for Molecular Diseases and Drug Research, International Center for Chemical and Biological Sciences, Karachi University, Karachi 75270, Pakistan

**Keywords:** epithelial ovarian cancer, cisplatin resistance, MalforminA1, apoptosis

## Abstract

High-grade epithelial ovarian cancer is a fatal disease in women frequently associated with drug resistance and poor outcomes. We previously demonstrated that a marine-derived compound MalforminA1 (MA1) was cytotoxic for the breast cancer cell line MCF-7. In this study, we aimed to examine the effect of MA1 on human ovarian cancer cells. The potential cytotoxicity of MA1was tested on cisplatin-sensitive (A2780S) and cisplatin-resistant (A2780CP) ovarian cancer cell lines using AlamarBlue assay, Hoechst dye, flow cytometry, Western blot, and RT-qPCR. MA1 had higher cytotoxic activity on A2780S (IC50 = 0.23 µM) and A2780CP (IC50 = 0.34 µM) cell lines when compared to cisplatin (IC50 = 31.4 µM and 76.9 µM, respectively). Flow cytometry analysis confirmed the cytotoxic effect of MA1. The synergistic effect of the two drugs was obvious, since only 13% of A2780S and 7% of A2780CP cells remained alive after 24 h of treatment with both MA1 and cisplatin. Moreover, we examined the expression of bcl2, p53, caspase3/9 genes at RNA and protein levels using RT-qPCR and Western blot, respectively, to figure out the cell death mechanism induced by MA1. A significant down-regulation in bcl2 and p53 genes was observed in treated cells compared to non-treated cells (*p* < 0.05), suggesting that MA1 may not follow the canonical pathway to induce apoptosis in ovarian cancer cell lines. MalforminA1 showed promising anticancer activity by inducing cytotoxicity in cisplatin-sensitive and cisplatin-resistant cancer cell lines. Interestingly, a synergistic effect was observed when MA1 was combined with cisplatin, leading to it overcoming its resistance to cisplatin.

## 1. Introduction

Ovarian cancer is one of the most common causes of death from cancer amongst women worldwide, and the second most common cause of death from gynecological cancers [1]. High-grade epithelial ovarian cancer (HG-EOC) accounts for 70–80% of all ovarian cancer histological subtypes. The vast majority of patients with HG-EOC are diagnosed with stage III/IV disease [2]. Cisplatin-based chemotherapy with or without anti-angiogenic therapy remains the standard of care [3,4,5]. However, the vast majority of patients experience a relapse of the disease [6]. A subset of patients with mutations in the BRCA1 or BRCA2 gene responds to inhibitors of the poly (ADP-ribose) polymerase (PARP) enzyme [7,8]. However, subsequently, the disease becomes resistant to cisplatin, and the 5-year survival rate for HG-EOC remains in the region of 15–45% [9]. Therefore, new compounds are urgently needed to achieve better control and overcome the resistance to frontline chemotherapy.

Marine drug research had evolved rapidly in the last decade, as marine organisms have been shown to acquire remarkable cytotoxic properties [10]. Several cytotoxic chemotherapeutic agents of marine origin, such as cytarabine, trabectedin, and eribulin, are in routine clinical use, and several others are in different stages of clinical trials [11,12,13,14]. In a recent study, Dobretsov et al. [15] screened 40 Omani marine natural products and extracts for anticancer activity using the MCF-7 breast cancer cell line. Of these, Malformin A1 (MA1) showed significant cytotoxicity. Three subtypes of Malformin have been identified, such as A, B, and C. MA1 is a cyclic pentapeptide produced by several marine fungi, including *Aspergillus niger* [16]. MA1 possesses a range of bioactive properties, including teratogenic, anti-bacterial, fibrinolytic, and cytotoxic activities [17,18]. Cytotoxicity of MA1 has been reported against human lung, pancreatic, breast, cervical, colorectal, and central nervous system cancer cell lines [19,20,21,22]. However, the cytotoxic activity of MA1 against ovarian cancer cell lines has not been studied.

This study aimed to examine whether MA1 has cytotoxic activity against human ovarian cancer cells, especially in comparison to cisplatin. Additionally, whether MA1 would be toxic against cisplatin-resistant cells and whether MA1 could overcome cisplatin resistance. Finally, we also aimed to study the mechanism of MA1-induced cytotoxicity.

## 2. Results

### 2.1. The Effect of Malformin A1 on Ovarian Cancer Cell Lines

**Figure 1:**
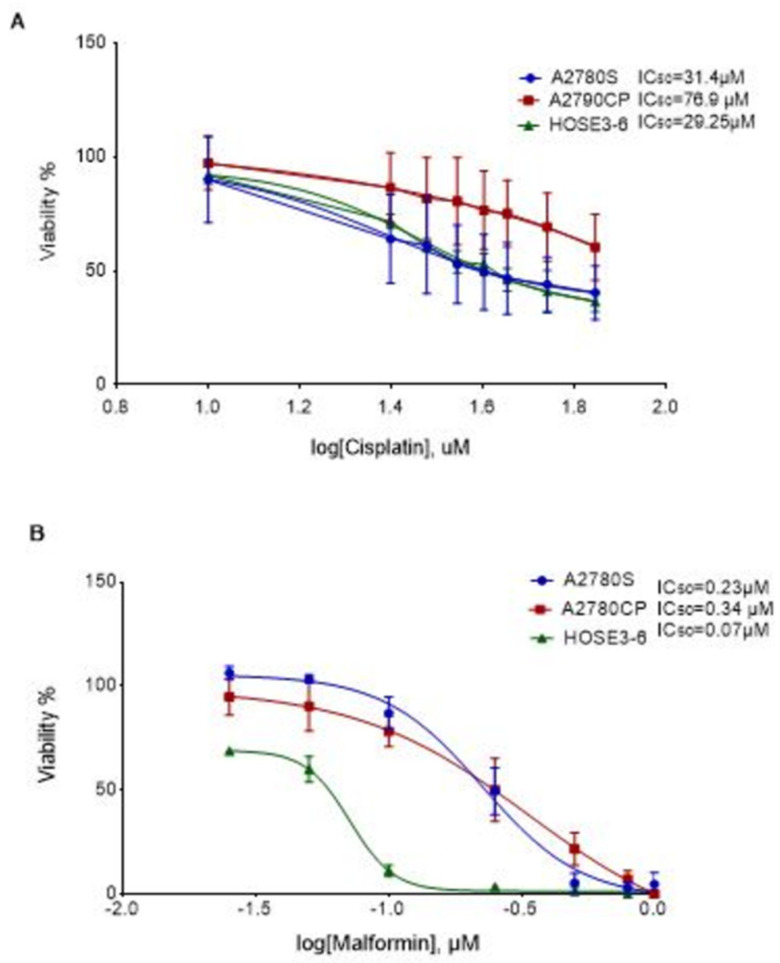
Effect of cisplatin and MA1 on the cell lines: (**A**) Viability percentage for A2780S and A2780CP in the presence of different doses of cisplatin. (**B**) Viability percentage for A2780S and A2780CP in the presence of different doses of Malformin A1. The control (no treatment) was set as 100% viability. The results are presented as mean + SE of three to five independent experiments.

The effect of MA1 on the viability of A2780S, A2780CP, and HOSE3-6 cells was evaluated using an Alamar Blue^TM^ viability assay. Increasing concentrations of MA1 resulted in a dose-dependent cell viability reduction in all cell lines. The normal ovarian cells, HOSE3-6, displayed the highest sensitivity to cisplatin and MA1 treatment. The effect of MA1 on cell viability (Figure 1B) was significantly more potent than the effect of cisplatin on the same cell lines (*p* < 0.001), (Figure 1A). 

### 2.2. Morphological Hallmarks of Cell Death

**Figure 2:**
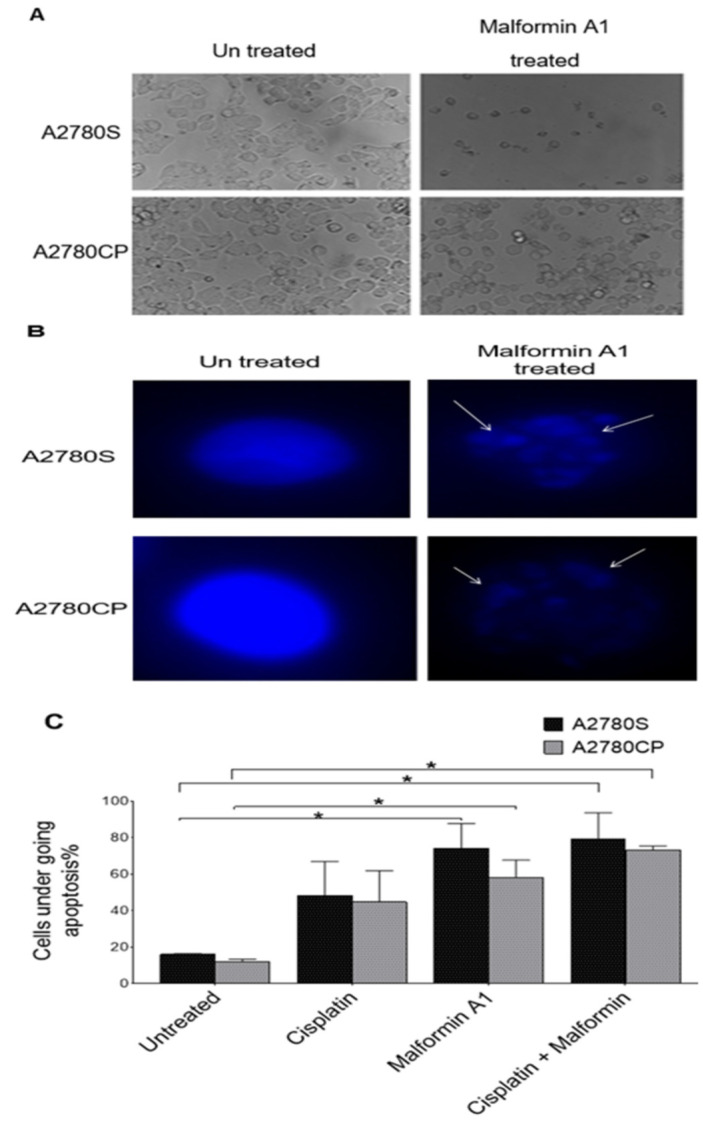
Cells’ morphology changes upon treatment with MA1: (**A**) Morphology of untreated ovarian cancer cell lines and Malformin A1 treated cells; (**B**) the nucleus major changes, as visualized by fluorescent microscopy following Hoechst stain using at 40× magnification. (**C**) The percentage of affected cells treated with MA1 compared to cisplatin treatments. Manual counts were performed blindly by three experienced observers on randomly selected fields containing at least 200 cells. * *p* < 0.05.

Microscopic examination of cells revealed significant morphological changes after treatment with cisplatin and/or MA1 (Figure 2A). Untreated ovarian cancer cells and MA1-treated and stained with the blue fluorescence Hoechst dye were used as indicators of unhealthy/dying cells (Figure 2B). The blue fluorescence stain in MA1-treated cells and cells treated with both compounds indicated highly condensed chromatin, which is characteristic of cells undergoing apoptosis (*p* = 0.008). The estimated percentage of the affected ovarian cancer cells after treatment with MA1 and cisplatin is displayed (Figure 2C).

### 2.3. The Effect of Combined Malformin A1 to Cisplatin

**Figure 3:**
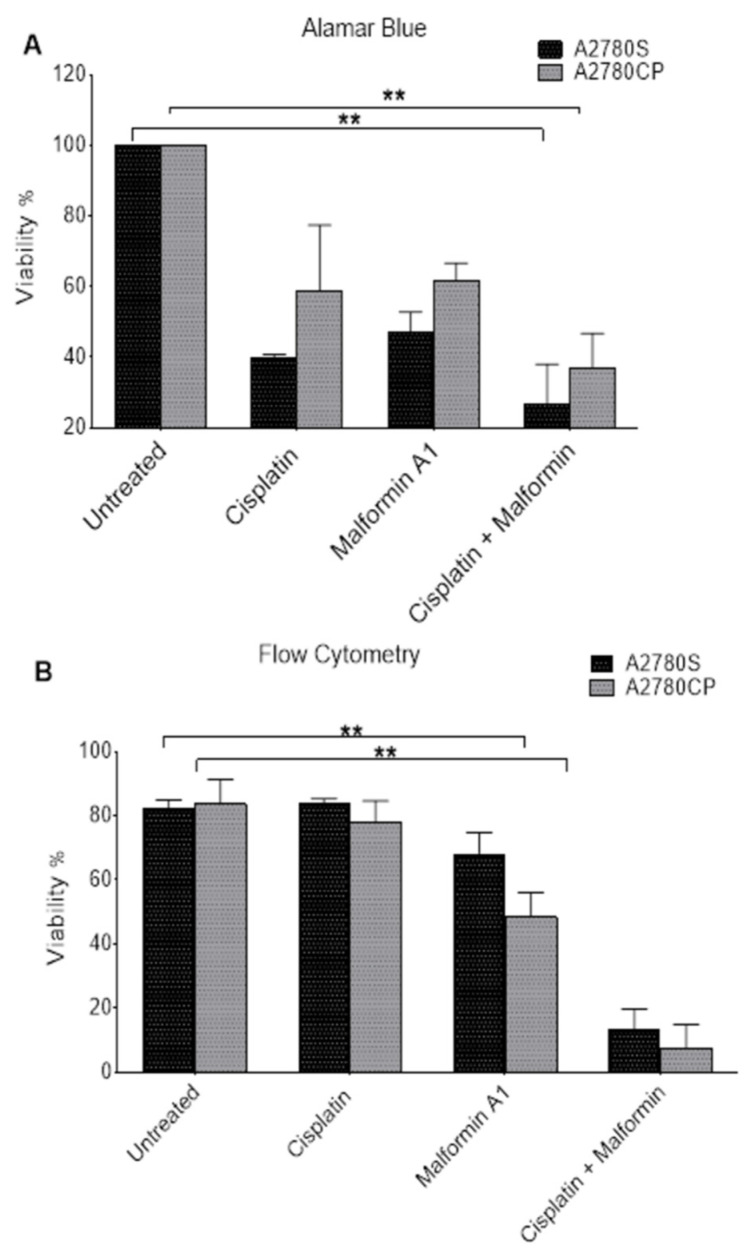
Cell survival assessment following treatment. The effect of combining cisplatin and MA1 on cisplatin-sensitive and cisplatin-resistant cells using Alamar Blue (**A**) and flow cytometry (**B**). The cells were treated with IC50 of cisplatin and Malformin A1 for 24 h. ** *p* < 0.01.

**Figure 4:**
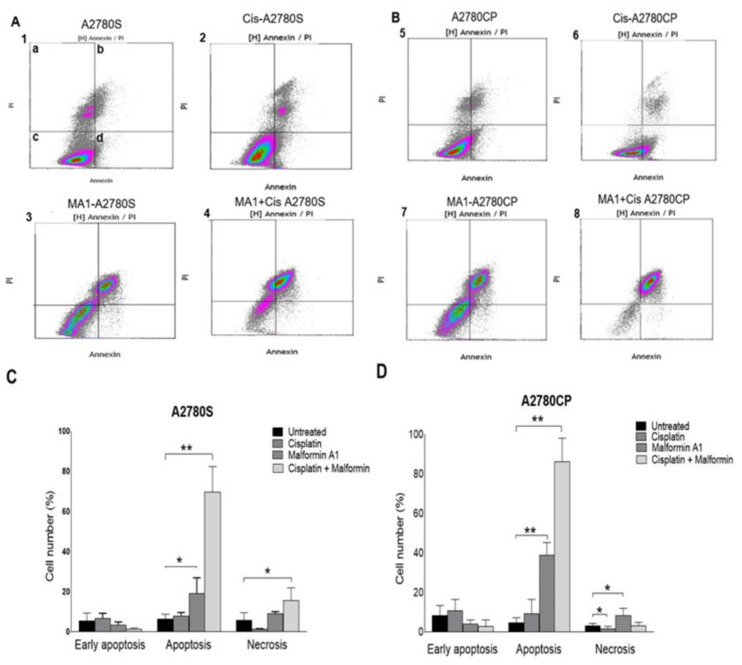
Flow cytometry analysis of A2780S and A2780CP cells after treatment with MA1 and cisplatin drugs. Effect of cisplatin and MA1 on A2780S cells (**A**), and A2780CP cells (**B**) assessed by flow cytometry. Ovarian cancer cells (5 × 10^5^) were treated with cisplatin and MA1 at a concentration equal to their corresponding IC_50_. Cells were treated with cisplatin (A2 and B6), MA1 (A3 and B7), or both (A4 and B8) for 24 h. Cell death was determined by flow cytometry using PI and Annexin dyes. (d) The number of early apoptotic cell deaths (annexin-V positive cells with PI negative), and (b) the late apoptotic cell deaths (annexin-V and PI-positive). (a) The number of necrotic cells (PI-positive, annexin-V negative). The percentage of proapoptotic, apoptotic, and necrotic (**C**) A2780S and (**D**) A2780CP cells after treatment with IC_50_ of cisplatin and Malformin A1 for 24 h. The results are presented as mean + SE of three independent experiments. * *p* < 0.05, ** *p* < 0.001.

The effect of MA1 on the viability of A2780S and A2780CP ovarian cancer cell lines was assessed by flow cytometry. Both Alamar Blue (Figure 3A) and flow cytometry (Figure 3B) methodologies showed a significant difference in cell viability when treated with MA1 and cisplatin. Likewise, the flow cytometry data showed a significant difference in the cells’ death following treatment with MA1 alone or combined with cisplatin (Figure 4C). A flow cytometry dot-plot-figure showed the effect of both MA1 and cisplatin treatment. The cells’ death was assessed by staining with annexin V dye (Y-axis, FL1) and PI (X-axis, FL4). The cells that were negative for both of the stains were considered alive cells (Figure 4A(1c)), whereas the number of annexin-V positive cells with PI negative cells determined the identification of early apoptotic cell death (Figure 4A(1d)), and late apoptotic cell death was quantified by the number of annexin-V positive and PI-positive cells (Figure 4A(1b)). The cells that were PI-positive and annexin-V negative were considered necrotic cells (Figure 4A(1a)). Based on the flow cytometry results, MA1 and cisplatin were found to have a significant effect on cisplatin-sensitive (Figure 4(A4)) and cisplatin-resistant (Figure 4(B8)) ovarian cancer cells.

### 2.4. Cell Cycle

**Figure 5:**
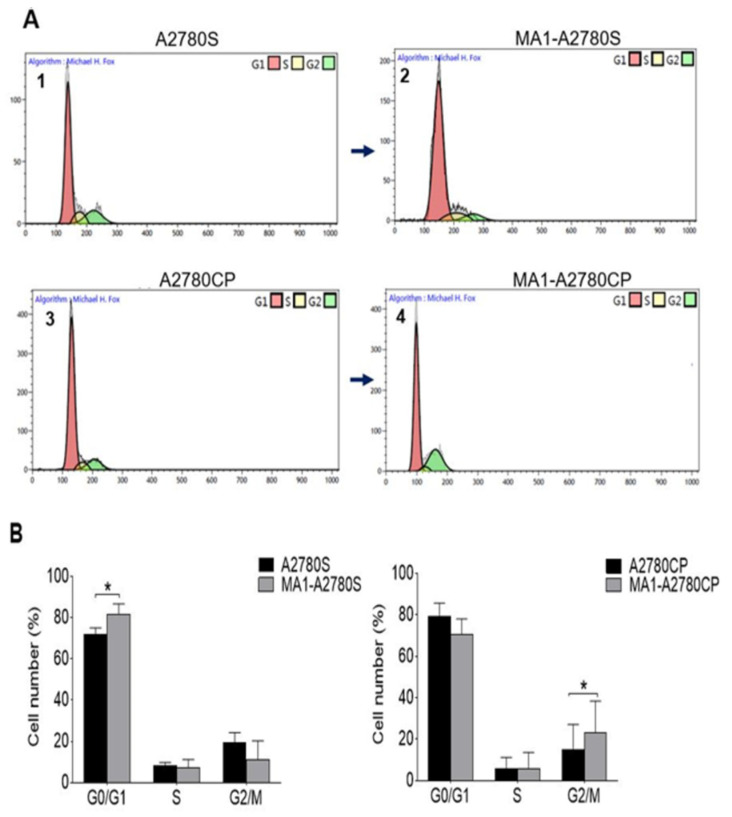
(**A**) Cell cycle analysis by flow cytometry based on propidium-iodide intercalation into the cellular chromatin. Data are presented as a relative fluorescence intensity in a 2-dimensional flow cytometry profile; (**1**) and (**3**) were untreated samples versus (**2**) and (**4**) treated samples for 24 h. (**B**) Comprehensive bar diagram of subpopulations of G0/G1, S, and G2/M. Ten thousand events were analyzed for each sample. Data represent the mean ± SE of three experiments at each concentration. * *p* < 0.05.

The cell cycle status was analyzed following a 24 h cell culture of the ovarian cancer cell lines. Cells’ distribution in G_0_/G_1_, S, and G_2_/M phases was estimated to be 71.8%, 8.3%, and 19.7% for the A2780S cell line, and 82.8%, 8.35%, and 8.75 for the resistance cell line, respectively. Following treatment with MA1, the cells’ number increased in the G_0_/G_1_ (80.5%) and G_2_/M (23%) phases for sensitive (A2780S) and resistant cell lines (A2780CP), respectively (Figure 5). The acquired data showed a different effect of MA1 in the distribution of the cell cycle phases between cisplatin-sensitive and cisplatin-resistance cell lines, suggesting that MA1-induced cytotoxicity occurs through different mechanisms.

### 2.5. Expression of Apoptosis-Related Genes in Ovarian Cancer Cells

**Figure 6:**
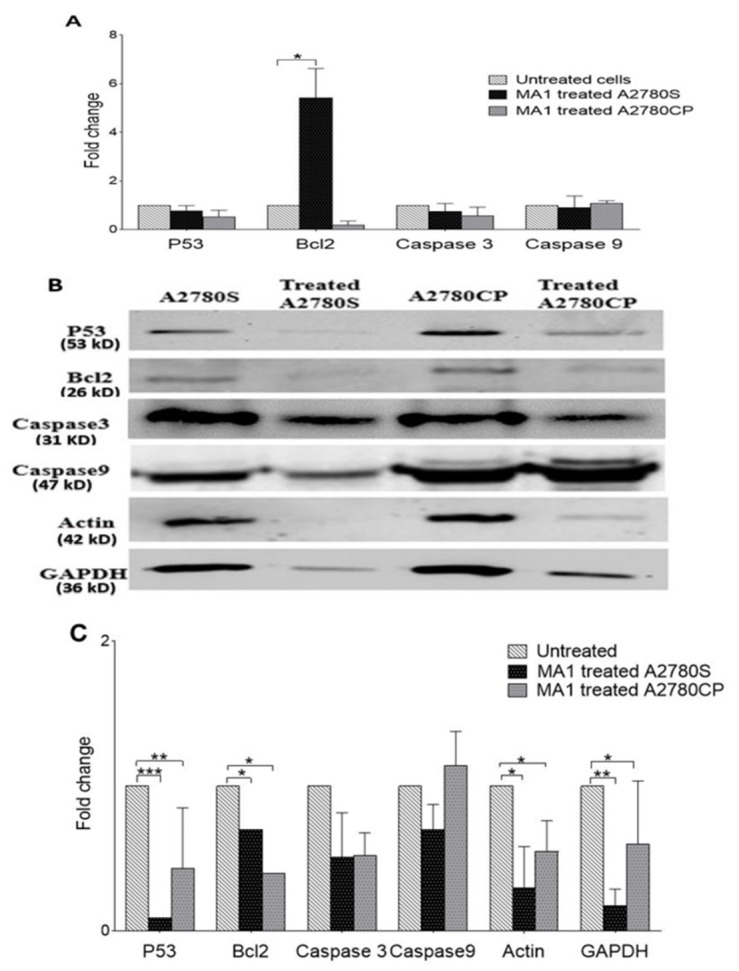
Expression of p53, Bcl2, and caspases3/9 genes’ analysis using quantitative RT-PCR and Western blot analysis: (**A**) RNA expression of p53, Bcl2, and caspases3/9 normalized to GAPDH. Protein expression of several genes in the ovarian cancer cells A2780S and A2780CP when treated with Malformin A1 for 24 h (**B**,**C**). The protein bands were normalized to the total load of protein using Coomassie blue staining. Data represent the mean ± SE of three experiments at each concentration. * *p* < 0.05. ** *p* < 0.01. *** *p* < 0.001.

RNA expression of the P53, Bcl2, and caspases3/9 genes was examined using the qRT-PCR technique. Differences in the gene expression were calculated as fold changes, and cycle threshold (Ct) values were used to compare the transcript quantity of the selected genes in different samples. MA1-treated A2780S cells showed a different RNA profile than MA1-treated A2780CP, in which two of the genes (Bcl2 and Caspases9) showed different expression patterns in the sensitive ovarian cancer cells upon treatment (Figure 6A). 

### 2.6. The Effect of MA1 Treatment on Proteins Expression

We further investigated the effect of MA1 on apoptosis-related protein expression. Caspase 3/9, Bcl2, and P53 protein expressions were examined and compared to the treated ovarian cancer cell lines using Western blotting. In A2780S- and A2780CP-treated cells, Bcl2 and P53 protein expression was markedly reduced, while Caspase 3/9 expression did not show significant changes in A2780S- and A2780CP-treated cells (Figure 6B,C). In addition, actin and GAPDH protein expression was significantly reduced following treatment; therefore, normalization was made by total protein Coomassie-blue stain (Figure S1 in Supplementary Materials).

## 3. Discussion

This is the first study reporting on the cytotoxicity of MA1 in ovarian cancer cell lines. MA1 was cytotoxic for the A2780S and the A2780CP ovarian cancer cell lines at less than micro-molar concentrations in a dose-dependent manner, and at significantly smaller concentrations than cisplatin. The addition of MA1 to cisplatin produced even more toxicity, especially in the cisplatin-resistant cell line, suggesting a synergistic activity of MA1 with cisplatin. The expression of the selected genes of the apoptotic and anti-apoptotic pathways suggested apoptosis as one mechanism of inducing cytotoxicity. However, MA1 may produce cytotoxicity not only by apoptosis, but also through other pathways. 

The cytotoxic effect of MA1 was tested in A2780S and A2780CP ovarian cancer cell lines using Alamar Blue, and DNA fragmentation was assessed by Hoechst stain. MA1 was found to be toxic against cisplatin-sensitive and cisplatin-resistant cell lines, with an IC50 of 0.23 and 0.34 µM, respectively. The corresponding IC50 of cisplatin in A2780S and A2780CP cells was estimated to be 31.4 µM and 76.9 µM, respectively, suggesting that MA1 was 136 times more potent than cisplatin in the cisplatin-sensitive cells, and 226 more potent in the cisplatin-resistant cells. In previous studies, the cytotoxicity of MA1 at a low concentration was reported using cervical cell lines. In HeLa cells, MA1 was cytotoxic at IC50 = 0.094 µM, as a result of the epigenetic changes introduced by MA1 in histone proteins [23]. In PC3 and LNCap prostate cancer cell lines, MA1 induced cell death through several pathways at an IC50 = 0.13 and 0.09 µM, respectively [21]. Moreover, a potent cytotoxicity of MA1 was reported in SW480 and DKO1 colorectal cancer cell lines, where caspase 3, 7, and 9 expressions were induced, and cell migration and invasion were suppressed through the stimulation of the p38 signaling pathway [20]. 

In order to confirm the cytotoxicity of MA1, we performed flow cytometry using Annexin V and PI dyes. The data revealed that a combination of MA1 and cisplatin caused higher cytotoxicity in A2780S (*p* = 0.005) and A278CP (*p* = 0.0001) cell lines, as compared to cisplatin treatment alone. Similar observations were recorded using the Alamar Blue and Hoechst stain methods. These results suggest that MA1 can be used as a potential combined therapy in ovarian cancer treatment. Indeed, several studies have reported the mechanisms underlying the acquired resistance to platinum chemotherapy, and combining MA1 with platinum would be a crucial step for assessing the synergistic effect and the likelihood of overcoming resistance. Different therapeutic strategies are under active research to overcome this problem, including the use of combined therapies [24]. 

Moreover, MA1 treatment tended to increase the proportion of sensitive ovarian cancer cells in the G_0_/G_1_ phase of the cell cycle. This is in agreement with what was proposed previously about sensitizing cancer cells to anticancer reagents using Malformin A and C, in which MA1 was found to evoke bleomycin-induced G_2_ arrest, resulting in an increase in sub G_1_ phase [19]. However, in the resistance cells, there was no change in G_0_/G_1_, although a slight significant increase was observed in G_2_/M after MA1 treatment. 

While protein expression analysis revealed a significant decrease in P53, Bcl2, ß-actin, and GAPDH in the MA1-treated ovarian cancer cells, caspase3 and caspase 9 protein expression status did not change significantly. These results suggest that the ovarian cancer cell death observed in this study was not executed through classical caspases activation. MA1 may introduce the cytotoxic effect through different mechanisms and pathways linked to cytoskeleton network. Indeed, a marked decrease in ß-actin and GAPDH expression was observed in the treated cells. Actin filament formation was reported to be under the control of LIMK gene, resulting in tumor growth prevention, invasion, and migration, suggesting a putative role of actin in cancer progression [25,26,27]. Likewise, GAPDH protein has been linked to numerous tumorigenic mechanisms and found to be up-regulated in different types of cancer [28,29,30]. The crucial function of GAPDH in the regulation of tubulin, one of the most important components of the cytoskeletal network, was reported in colon cancer cell lines [31]. In addition, our results showed different expression levels of Bcl2 RNA and protein in the ovarian cancer cells, pointing to the significant controlling role played by translational and post-translation mechanisms that have been observed in lymphoid tissues [32]. In fact, both P53 and Bcl2 genes seem to contribute to MA1-induced cytotoxicity, as indicated by our results. Mutations in the Tp53 are commonly reported in almost all types of cancers, including ovarian cancer [33]. Piling evidence indicated that Tp53 mutations promote resistance to anticancer treatments and contribute actively to tumor progression [34]. Likewise, Bcl2 expression was reported to improve the survival of cancerous cells [35,36]. Data obtained on caspase-3 can be explained by its putative role in cancer pathogenesis by sensitizing cancer cells to drug treatments. As suggested earlier, knockout/knockdown of caspase-3 in human colorectal cancer cells increased their sensitivity to DNA-damaging agents, such as 5-fluorouracil (5-FU), camptothecin, and etoposide in vivo and in vitro. Moreover, the effect of caspase-3 deficiency in sensitizing tumors promoted the caspase-3 inhibitors’ use as an adjuvant in cancer radiotherapy [37]. These findings suggest a potential role of caspase3 in chemosensitizing cancer cells to therapeutic agents [38].

## 4. Materials and Methods

### 4.1. Reagents

Malformin A1 was purchased from Boc Science Shirley, NY, USA) and dissolved in dimethyl sulfoxide (DMSO) (Sigma, St. Louis, Missouri, MO, USA) to make a stock solution of 1 mg/mL. cisplatin was purchased from Mylan (PAR, Paris, France) as a 50 mg/mL solution.

### 4.2. Cell Culture

The cisplatin-sensitive (A2780S) and cisplatin-resistant (A2780CP) ovarian cancer cell lines were a generous gift from Dr. Benjamin Tsang. Professor GSW Tsao from Hong Kong University provided immortalized normal ovarian cell line HOSE3-6. A2780S and A2780CP cells were propagated in DMEM/F12 (Gibco, New York, NY, USA) media, while HOSE3-6 cells were grown in MEM (Gibco, New York, NY, USA) at 37 °C in a humidified atmosphere with 5% CO2. All culture media were supplemented with 10% fetal bovine serum (FBS) (Gibco, New York, NY, USA) and 1% penicillin/streptomycin (Gibco, New York, NY, USA). Cells were subjected to different concentrations of MA1 (0.025–1 μM) and cisplatin (10–70 μM) for 24 h in order to examine the anticancer effects of these drugs.

### 4.3. Cell Viability Assay

Cell viability was determined using Alamar Blue reagent (Invitrogen, Waltham, MA, USA). A2780S, A2780CP, and HOSE3-6 cell lines were seeded in 96-well culture plates at a density of 104 cells per well and grown in a complete culture media (DMEM-F12/MEM + 10% FBS) at 37 °C and 5% CO2 for 24 h. The medium was then replaced with serum-free media (plain media), and different concentrations of MA1 (0.025–1 μM) and cisplatin (10–70 μM) were applied. Plain media alone was used as a negative control for cisplatin, while plain media containing DMSO was used as a negative control for Malformin A1. Cell proliferation was monitored by adding Alamar Blue (10% of the final volume) to the culture medium and incubating for 3 h at 37 °C. The absorbance was measured at 570nm using a Multiscan spectrum spectrophotometer (Thermo Fisher Scientific Inc., Waltham, MA, USA).

Each experiment was performed in triplicate and repeated three times. The percentage of cell viability was calculated according to the following equation:

$$\frac{\mathrm{mean}\;\mathrm{OD}\;\mathrm{of}\;\mathrm{treated}\;\mathrm{cell}\;-\mathrm{mean}\;\mathrm{OD}\;\mathrm{of}\;\mathrm{blank}}{\mathrm{mean}\;\mathrm{OD}\;\mathrm{of}\;\mathrm{untreated}\;\mathrm{cell}\;\left(\mathrm{negative}\;\mathrm{control}\right)-\mathrm{mean}\;\mathrm{OD}\;\mathrm{of}\;\mathrm{blank}}\;\times \;100$$

### 4.4. Hoechst Dye Staining of Cells

A total of 1 × 104 cells (A2780S, A2780CP, HOSE3-6) were plated in 24-well plates and cultured for 24 h. Then, the media was replaced with serum-free media, and the cells were treated with the IC50 concentration of MA1 and cisplatin for 24 h. Plain media was used as a negative control for cisplatin, and plain media containing DMSO was used as a negative control for Malformin A1. Then, cells were detached using 2 mM EDTA, washed with plain media, and centrifuged for 5 min (100 × rcf). The supernatant was removed, and cells were fixed and stained in 10% formalin containing 6.25 ng/mL of Hoechst 33258 dye before microscopic examination using Nikon Eclipse Ti-S fluorescence microscope (40× magnification). Manual counts were performed blindly by three experienced observers on randomly selected fields containing at least 200 cells.

### 4.5. Annexin V-FITC Apoptosis and Cell Cycle Assays

Flow cytometry was used to study cell apoptosis and cell cycle after treatment with different compounds. Cells (5 × 105/well) were plated in 6-well plates and incubated for 24 h in the presence or absence of MA1. An apoptosis assay was performed using Annexin V-FITC Apoptosis Detection Kit (PN IM3546, Beckman Coulter, Indianapolis, IN, USA), while cell cycle reagent (C03551, Beckman Coulter, Indianapolis, IN, USA) was used to assess the cell cycle distribution pattern for A2780S and A2780CP cells, according to the manufacturer’s protocol. Briefly, cells were detached using 0.05% Trypsin/EDTA (Gibco, New York, NY, USA) and, for apoptosis analysis, Annexin V and PI dyes were added and incubated in the dark on ice for 20 min. In the cell cycle, cells were harvested and immediately fixed in 70% (*v/v*) ethanol overnight at 4 °C, then stained with PI reagent. The data were acquired using Navios flow cytometry (Beckman coulter Life Sciences, Indianapolis, IN, USA) and analyzed using Kaluza software 2.1 (Beckman coulter Life Sciences, Indianapolis, IN, USA).

### 4.6. Quantitative Real Time-PCR (qPCR)

Total RNA was extracted from A2780S, A2780CP, and HOSE3-6 cells using the PureLink RNA mini Kit (Invitrogen, Waltham, MA, USA), as described by the manufacturer, and subsequently reverse-transcribed to complementary DNA using a High Capacity cDNA Reverse Transcription kit (Applied Biosystems™, Waltham, MA, USA). We used TaqMan pre-optimized probes to perform qRT-PCR on an ABI 7500 Fast real-time PCR machine (Applied Biosystems, Austin, TX, USA). Data were normalized to GAPDH expression, and relative expression was calculated by the comparative Ct method (ΔΔCt).

### 4.7. Western Blot Analysis

Cells were cultured in a 25 cm flask and treated with the IC50 concentration of MA1 24 h before harvest. Cells were then lysed in lysis buffer (50 mM Tris-HCl pH 7.4, 1% NP-40, 0.25% sodium deoxycholate, 0.1% SDS, 150 mM NaCl, 1 mM EDTA, proteases, and phosphatases inhibitors) and centrifuged for 15 min at 13000 rpm in a cold room (5 °C).

Protein concentration was determined by the Bradford test using Bio-Rad protein reagent assay. Then, 30–50 µg of proteins were separated by SDS-PAGE and transferred to a PVDF/nitrocellulose membrane. The membrane was blocked in 5% bovine serum albumin (BSA) in TBS-T and probed overnight with antibodies (Santa Cruz Biotechnology, Dallas, TX, USA) directed against the proteins to be analyzed (e.g., p53, Bcl2, caspase3, caspase9, actin, and GAPDH). Primary antibodies were used at a dilution of 1/200 in the blocking reagent. After a washing step of 3 × 5 min in TBS-T, membranes were incubated at room temperature for two hours with HRP-conjugated secondary IgG antibodies (Thermoscientific, Waltham, MA, USA) at a 1:5000 dilution. Membranes were revealed using ECL Western blotting detection reagent kit (Pierce Biotechnology, Rockford, IL, USA).

### 4.8. Statistical Analysis

Statistical analyses were performed using Statistical Package for the Social Sciences (SPSS, Version 23) and the GraphPad Prism (version 8.1.2 GraphPad Software Inc., San Diego, CA, USA). One-Way ANOVA, Friedman tests, as well as median inhibition concentration IC50, were performed. The statistical significance was set at *p* < 0.05 (*), *p* < 0.01 (**), *p* < 0.001 (***) and *p* < 0.0001 (****).

## 5. Conclusions

In conclusion, our results suggest MA1 as a potential anticancer agent for ovarian cancer treatment. It induced cytotoxicity both in cisplatin-sensitive and cisplatin-resistant cancer cell lines at micromolar concentrations. Moreover, the addition of MA1 to cisplatin produced a synergistic effect. MA1 induces cytotoxicity through different mechanisms, including apoptosis. Further investigations to reveal MA1’s exact mechanism of action are ongoing and could help in the treatment of ovarian cancer. The compound merits further investigation for the management of high-grade epithelial ovarian cancer.

## Data Availability

The data presented in this study are available in Supplementary Materials.

## Supplementary Materials

Figure S1: Total proteins load by Coomassie brilliant blue staining of SDS-polyacrylamide gel. Different protein lysates from cancer cells (50 mg/mL) were loaded in 8% SDS-polyacrylamide gel and run for approximately 2 h. PageRuler Prestained Protein Ladder (Thermo Fhisher, Waltham, MA, USA) was used as molecular size marker.
