# Supplementary material for: Malformin-A1 (MA1) Sensitizes Chemoresistant Ovarian Cancer Cells to Cisplatin-Induced Apoptosis
Source: Molecules. 2021 Jun 13;26(12):3624. doi: 10.3390/molecules26123624 (PMC8231817; doi:10.3390/molecules26123624)
Supplement: Supplementary file 1 [file molecules-26-03624-s001.zip › molecules-1233210-supplementary.pdf]

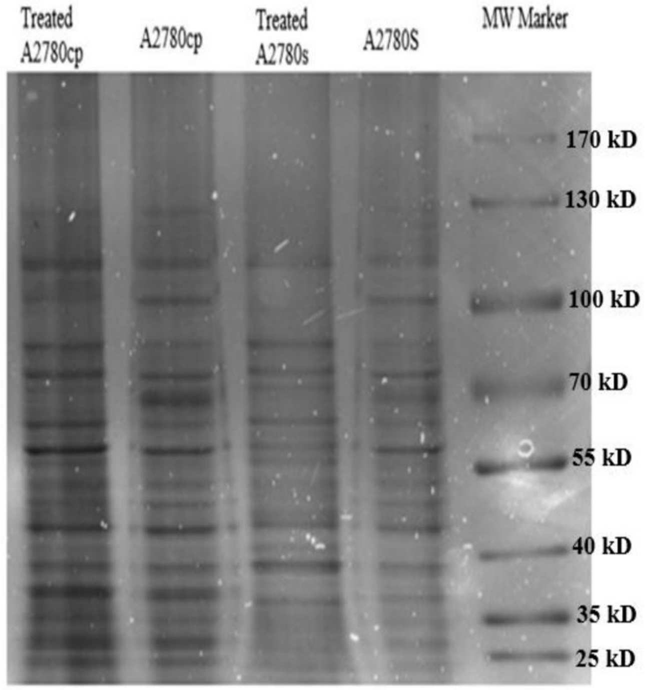

Figure S1: Total proteins load by Coomassie brilliant blue staining of SDS-polyacrylamide gel. Different protein lysates from cancer cells (50 mg/mL) were loaded in 8% SDS-polyacrylamide gel and run for approximately 2 h. PageRuler Prestained Protein Ladder (Thermo Fhisher, USA) was used as molecular size marker.
